# Prospective and External Evaluation of a Machine Learning Model to Predict In-Hospital Mortality of Adults at Time of Admission

**DOI:** 10.1001/jamanetworkopen.2019.20733

**Published:** 2020-02-07

**Authors:** Nathan Brajer, Brian Cozzi, Michael Gao, Marshall Nichols, Mike Revoir, Suresh Balu, Joseph Futoma, Jonathan Bae, Noppon Setji, Adrian Hernandez, Mark Sendak

**Affiliations:** 1 Duke Institute for Health Innovation, Durham, North Carolina; 2 Duke University School of Medicine, Durham, North Carolina; 3 Department of Statistical Science, Duke University, Durham, North Carolina; 4 John A. Paulson School of Engineering and Applied Sciences, Harvard University, Boston, Massachusetts; 5 Department of Medicine, Duke University School of Medicine, Durham, North Carolina

## Abstract

**Importance:**

The ability to accurately predict in-hospital mortality for patients at the time of admission could improve clinical and operational decision-making and outcomes. Few of the machine learning models that have been developed to predict in-hospital death are both broadly applicable to all adult patients across a health system and readily implementable. Similarly, few have been implemented, and none have been evaluated prospectively and externally validated.

**Objectives:**

To prospectively and externally validate a machine learning model that predicts in-hospital mortality for all adult patients at the time of hospital admission and to design the model using commonly available electronic health record data and accessible computational methods.

**Design, Setting, and Participants:**

In this prognostic study, electronic health record data from a total of 43 180 hospitalizations representing 31 003 unique adult patients admitted to a quaternary academic hospital (hospital A) from October 1, 2014, to December 31, 2015, formed a training and validation cohort. The model was further validated in additional cohorts spanning from March 1, 2018, to August 31, 2018, using 16 122 hospitalizations representing 13 094 unique adult patients admitted to hospital A, 6586 hospitalizations representing 5613 unique adult patients admitted to hospital B, and 4086 hospitalizations representing 3428 unique adult patients admitted to hospital C. The model was integrated into the production electronic health record system and prospectively validated on a cohort of 5273 hospitalizations representing 4525 unique adult patients admitted to hospital A between February 14, 2019, and April 15, 2019.

**Main Outcomes and Measures:**

The main outcome was in-hospital mortality. Model performance was quantified using the area under the receiver operating characteristic curve and area under the precision recall curve.

**Results:**

A total of 75 247 hospital admissions (median [interquartile range] patient age, 59.5 [29.0] years; 45.9% involving male patients) were included in the study. The in-hospital mortality rates for the training validation; retrospective validations at hospitals A, B, and C; and prospective validation cohorts were 3.0%, 2.7%, 1.8%, 2.1%, and 1.6%, respectively. The area under the receiver operating characteristic curves were 0.87 (95% CI, 0.83-0.89), 0.85 (95% CI, 0.83-0.87), 0.89 (95% CI, 0.86-0.92), 0.84 (95% CI, 0.80-0.89), and 0.86 (95% CI, 0.83-0.90), respectively. The area under the precision recall curves were 0.29 (95% CI, 0.25-0.37), 0.17 (95% CI, 0.13-0.22), 0.22 (95% CI, 0.14-0.31), 0.13 (95% CI, 0.08-0.21), and 0.14 (95% CI, 0.09-0.21), respectively.

**Conclusions and Relevance:**

Prospective and multisite retrospective evaluations of a machine learning model demonstrated good discrimination of in-hospital mortality for adult patients at the time of admission. The data elements, methods, and patient selection make the model implementable at a system level.

## Key Points

**Question:**

How accurately can a machine learning model predict risk of in-hospital mortality for adult patients when evaluated prospectively and externally?

**Findings:**

In this prognostic study that included 75 247 hospitalizations, prospective and multisite retrospective evaluations of a machine learning model demonstrated good discrimination in predicting in-hospital mortality for patients at the time of admission. Area under the receiver operating characteristic curve ranged from 0.84 to 0.89, and prospective and multisite retrospective results were similar.

**Meaning:**

A machine learning model, designed to be implementable at a system level, demonstrated good discrimination in identifying patients at high risk of in-hospital mortality and may be used to improve clinical and operational decision-making.

## Teaser

This prognostic study evaluates the discrimination of a machine learning model to predict in-hospital mortality for all adult patients at the time of hospital admission.

## Introduction

Approximately 2% of patients admitted to US hospitals die during the inpatient admission.^1^ Efforts to reduce preventable in-hospital mortality have focused on improving treatments and care delivery,^2^ and efforts to reduce nonpreventable mortality have focused on supporting patient preferences to die at home^3,4,5^ and attempting to reduce health care costs in the inpatient setting. Early identification of patients at high risk of in-hospital mortality may improve clinical and operational decision-making and improve outcomes for patients.^6^

Previously developed machine learning models that predict in-hospital mortality have not been evaluated prospectively,^7,8,9,10,11^ and most have not been externally validated.^7,8,9,10^ As a result, there is no clear evidence that these models would alter clinical practice or improve clinical outcomes. However, prior models developed to predict deterioration and intensive care unit (ICU) transfer have been evaluated retrospectively and implemented at various health systems.^12,13,14,15^ Additionally, early pilot data suggest the potential for machine learning models predicting mortality to influence physician decision-making.^16^ Prospectively evaluating the performance of machine learning models run on real-world, operational data is a crucial step toward integrating these models into the clinical setting and evaluating the impact on patient care. External validation on patient data from distinct geographic sites is needed to understand how models developed at one site can be safely and effectively implemented at other sites.^17^

Unfortunately, formidable barriers prevent prospective and external evaluation of machine learning models and their integration into clinical care. First, after developing a model on retrospective data, significant investment is needed to integrate the model into a production electronic health record (EHR) system.^18^ Second, most in-hospital mortality models are disease- or department-specific and only evaluated on local data, limiting the ability to scale the benefit that can be realized from a single model.^19,20,21,22,23^ Third, many efforts to date use either proprietary data sets or advanced, computationally intensive modeling methods,^7^ which limit adoption owing to the technical capabilities required for successful integration.

The primary aim of this study was to prospectively and externally validate a machine learning model that predicts in-hospital mortality at the time of hospital admission for adult patients. The model uses readily available EHR data and accessible computational methods, can be applied to all adult patients, and was designed to be integrated into clinical care to support workflows that address the needs of patients at high risk of mortality. We evaluated the model on retrospective data from 3 hospitals and prospectively on a technology platform integrated with the production EHR system.

## Methods

### Setting

This study was performed at Duke University Health System, a quaternary, academic, 3-site hospital system with 1512 beds that had approximately 69 000 inpatient admissions in fiscal year 2018. It adopted an Epic Systems EHR system in 2013. Both the retrospective and prospective evaluations were approved by the Duke University Health System institutional review board, which waived the need for informed consent for the use of identifiable data. This study adheres to the Transparent Reporting of a Multivariable Prediction Model for Individual Prognosis or Diagnosis (TRIPOD) reporting guideline for diagnostic and prognostic studies.

### Model Training and Development

We trained the model using EHR data from a total of 43 180 hospitalizations representing 31 003 unique adult patients admitted to a quaternary academic hospital (hospital A) from October 1, 2014, to December 31, 2015. The outcome label of in-hospital mortality was defined as a discharge disposition of “expired.” A total of 195 model features were built using 57 EHR data elements, which included patient demographic characteristics (5 data elements), laboratory test results (33 data elements), vital signs (9 data elements), and medication administrations (10 data elements) that occurred between presentation to the hospital and the time of inpatient admission. As the objective was to build a model that performed best on the prediction task, feature selection was not performed on the data set. Time of inpatient admission was defined as time of order placement to admit a patient. All hospitalizations of adult patients with an order placed for inpatient admission were included. Patients who died in the emergency department after an order was placed for admission but before the patient was physically moved to an inpatient room were included. The laboratory test results and vital signs were aggregated by taking the minimum, maximum, mean, and variance of the recorded values, and the medications were aggregated by the count of each medication type. The data elements and model features are provided in eFigure 1 in the Supplement.

Missing values for each variable were assigned a default value of “NA” (not available), which served as an indicator for missingness. This allowed the algorithm to include observations with missing features and to gain signal from missingness itself. This missingness designation allows the model to account for situations in which the presence or absence of a preadmission measurement (eg, blood glucose level) may be indicative of an underlying patient feature (eg, diabetes). Other studies have also shown that the XGBoost model (The XGBoost Contributors) can gain signal from missingness (or nonmissingness) without resorting to imputation techniques.^24^

We randomly selected 75% of encounters for model training and held out 25% for testing. Initial tests demonstrated superior performance of gradient-boosting models compared with random forest and regression models, so we selected a gradient-boosting model using the Python programming language (Python Software Foundation) with the XGBoost package^24,25^ as our base model, with parameters chosen using cross-validation. We used the CalibratedClassifierCV^26^ in Scikit-learn (The Scikit-learn Contributors)^27^ with 10 cross-validation folds to calibrate the output so that the predictions would better correspond to observed probabilities.

For each iteration of the cross-validation, the model fit the 9 training folds with the XGBoost model. This fitted model was then used to generate predictions for the holdout fold. These predictions were then calibrated using isotonic regression. From this procedure, we trained 10 models, each consisting of an XGBoost prediction step and an isotonic regression step. To generate a prediction on a new patient, we took the arithmetic mean of the output of these 10 models.

### Statistical Analysis

To assess model discrimination, the receiver operating characteristic (ROC) and the precision recall (PR) curves were plotted and the respective area under the ROC curve (AUROC) and area under the PR curve (AUPRC) were obtained. The AUPRC was included because it is particularly informative when evaluating binary classifiers on imbalanced data sets, in which the number of negative outcomes significantly outweighs the number of positive outcomes.^28,29^ Model calibration was assessed by comparing the predicted and empirical probability curves. We obtained 95% confidence intervals for the AUROC, AUPRC, and calibration plot by bootstrapping the observations of the test data. Model output was plotted against observed mortality rates to assess calibration. Additionally, to assess performance in a manner that is meaningful to operational stakeholders, sensitivity, specificity, positive predictive value (PPV), and number of true and false alarms per day were generated for various risk thresholds. Model performance was evaluated on different patient subpopulations, segmented by information missingness, age, race, sex, admission types, and admission sources. Sensitivity, specificity, and PPV were obtained for each subpopulation using a set risk threshold that resulted in a PPV of 20% across the entire population.

### Retrospective Validations

A separate cohort of 16 122 hospitalizations representing 13 094 unique adult patients admitted to this same quaternary academic hospital (hospital A) from March 1, 2018, to August 31, 2018, was used to assess temporal generalizability. Two separate cohorts from different community-based hospitals (hospital B and hospital C) were used to assess external generalizability. Hospital B had a cohort of 6586 hospitalizations representing 5613 unique adult patients and hospital C had a cohort of 4086 hospitalizations representing 3428 unique adult patients. Both external cohorts consisted of admissions between March 1, 2018, and August 31, 2018. The AUROC and AUPRC for these 3 validations are reported.

### Prospective Validation

The model was integrated into the production EHR system and prospectively validated at hospital A, the quaternary academic hospital on which the model was initially trained. The EHR report database is updated nightly at 12 am (midnight). All required data for the model for a given day are uploaded to the reporting database via an automated process that takes about 4 hours. The model runs after this process completes, at 5 am every morning. The delay between inpatient admission and risk score generation ranges from 29 hours for patients admitted at 12:01 am the prior day to 5 hours for patients admitted at 11:59 pm the prior day. The prospective cohort consisted of 5273 hospitalizations representing 4525 unique adult patients admitted between February 14, 2019, and April 15, 2019. The model was run daily on a pipeline that automatically curates EHR data,^30^ and the patient encounter identifier and risk scores were stored in a database over a 2-month period. Model output was not exposed to clinicians and was not used in clinical care during this silent period. At the end of the silent period, in-hospital mortality outcomes were obtained for each patient encounter. Model performance was assessed using methods identical to the retrospective validations. The AUROC and AUPRC for this prospective cohort were reported.

### Implementation

During the course of this study, we partnered with clinical and operational leaders to better understand how this model should be used in the hospital setting. As part of this work, we iteratively developed a simple user interface (eFigure 3 in the Supplement) showing model output using Apache Superset^31^ in addition to a workflow decision framework (eFigure 4 in the Supplement). We developed a model fact sheet, similar to an over-the-counter drug label, that clearly communicates important information about the model to clinician end users.

## Results

We included a total of 75 247 hospitalizations (median [interquartile range] patient age, 59.5 [29.0] years; 45.9% involving male patients) in the training, retrospective validation, and prospective validation of the model. The overall percentage of hospitalizations with in-hospital mortality was 2.7% (2021 hospitalizations). The hospital and encounter characteristics are summarized by training and testing cohorts in Table 1.

**Table 1.** Characteristics of Hospitalizations in Training and Test Data Sets

Characteristic	No. (%)				
	Training Data	Retrospective			Prospective
	Hospital A (2014-2015)	Hospital A (2018)	Hospital B (2018)	Hospital C (2018)	Hospital A (2019)
Admissions					
Hospitalizations, No.	43 180	16 122	6586	4086	5273
Length of stay, median, d	3.82	3.81	3.0	3.1	3.52
Demographic					
Age, median (IQR), y	58.6 (29.0)	60.1 (29.1)	59.0 (32.3)	66.0 (21.0)	59.7 (31.0)
Male	20 359 (47.1)	7375 (45.7)	2574 (39.1)	1906 (46.6)	2347 (44.5)
Discharge disposition					
Home	35 645 (82.5)	13 231 (82.1)	5215 (79.2)	3144 (76.9)	4537 (86.0)
Skilled nursing facility	3937 (9.1)	1576 (9.8)	736 (11.2)	541 (13.2)	487 (9.23)
Another health care facility	589 (1.4)	201 (1.2)	279 (4.2)	105 (2.6)	49 (0.89)
Rehabilitation	564 (1.3)	204 (1.3)	90 (1.4)	87 (2.1)	22 (.042)
Other	159 (0.4)	112 (0.7)	58 (0.9)	34 (0.8)	16 (0.30)
In-hospital death—primary outcome	1298 (3.0)	435 (2.7)	119 (1.8)	84 (2.1)	84 (1.6)

Abbreviation: IQR, interquartile range.

Table 2 summarizes the prediction accuracy by evaluation method, site, and time period. The in-hospital mortality rates for the training validation; retrospective validations at hospitals A, B, and C; and prospective validation cohorts were 3.0%, 2.7%, 1.8%, 2.1%, and 1.6%, respectively. For the retrospective validations, the AUROC was 0.87 (95% CI, 0.83-0.89) for the 25% held-out test portion of the original training data set (retrospective evaluation, hospital A, 2014-2015), 0.85 (95% CI, 0.83-0.87) for a temporal validation cohort (retrospective evaluation, hospital A, 2018), 0.89 (95% CI, 0.86-0.92) for an external temporal and geographic validation cohort (retrospective evaluation, hospital B, 2018), and 0.84 (95% CI, 0.80-0.89) for an additional external temporal and geographic validation cohort (retrospective evaluation, hospital C, 2018). For the prospective validation, the AUROC was 0.86 (95% CI, 0.83-0.90) (prospective validation, hospital A, 2019). The AUPRC, which compares sensitivity (recall) and PPV (precision) and is more dependent on prevalence of the outcome, was lower across the 2018 retrospective and 2019 prospective validations compared with the earlier 2014 to 2015 retrospective validation. The AUPRC for the training validation; retrospective validations at hospitals A, B, and C; and prospective validation cohorts were 0.29 (95% CI, 0.25-0.37), 0.17 (95% CI, 0.13-0.22), 0.22 (95% CI, 0.14-4 0.31), 0.13 (95% CI, 0.08-0.21), and 0.14 (95% CI, 0.09-0.21), respectively. The prevalence of the outcome decreased from 3.0% in the 2014 to 2015 training data set compared with 1.6% in the prospective validation cohort. In practice, this means that at a fixed sensitivity of 60%, the PPV of the model in the 2014 to 2015 cohort was 12%, compared with 8.5% in the prospective validation cohort.

**Table 2.** Prediction Accuracy by Evaluation Method, Location, and Time

Evaluation Method	Location	Time	AUROC (95% CI)	AUPRC (95% CI)
Retrospective	Hospital A	2014-2015	0.87 (0.83-0.89)	0.29 (0.25-0.37)
Retrospective	Hospital A	2018	0.85 (0.83-0.87)	0.17 (0.13-0.22)
Retrospective	Hospital B	2018	0.89 (0.86-0.92)	0.22 (0.14-0.31)
Retrospective	Hospital C	2018	0.84 (0.80-0.89)	0.13 (0.08-0.21)
Prospective	Hospital A	2019	0.86 (0.83-0.90)	0.14 (0.09-0.21)

Abbreviations: AUPRC, area under the precision recall curve; AUROC, area under the receiver operating characteristic curve.

**Figure 1.**
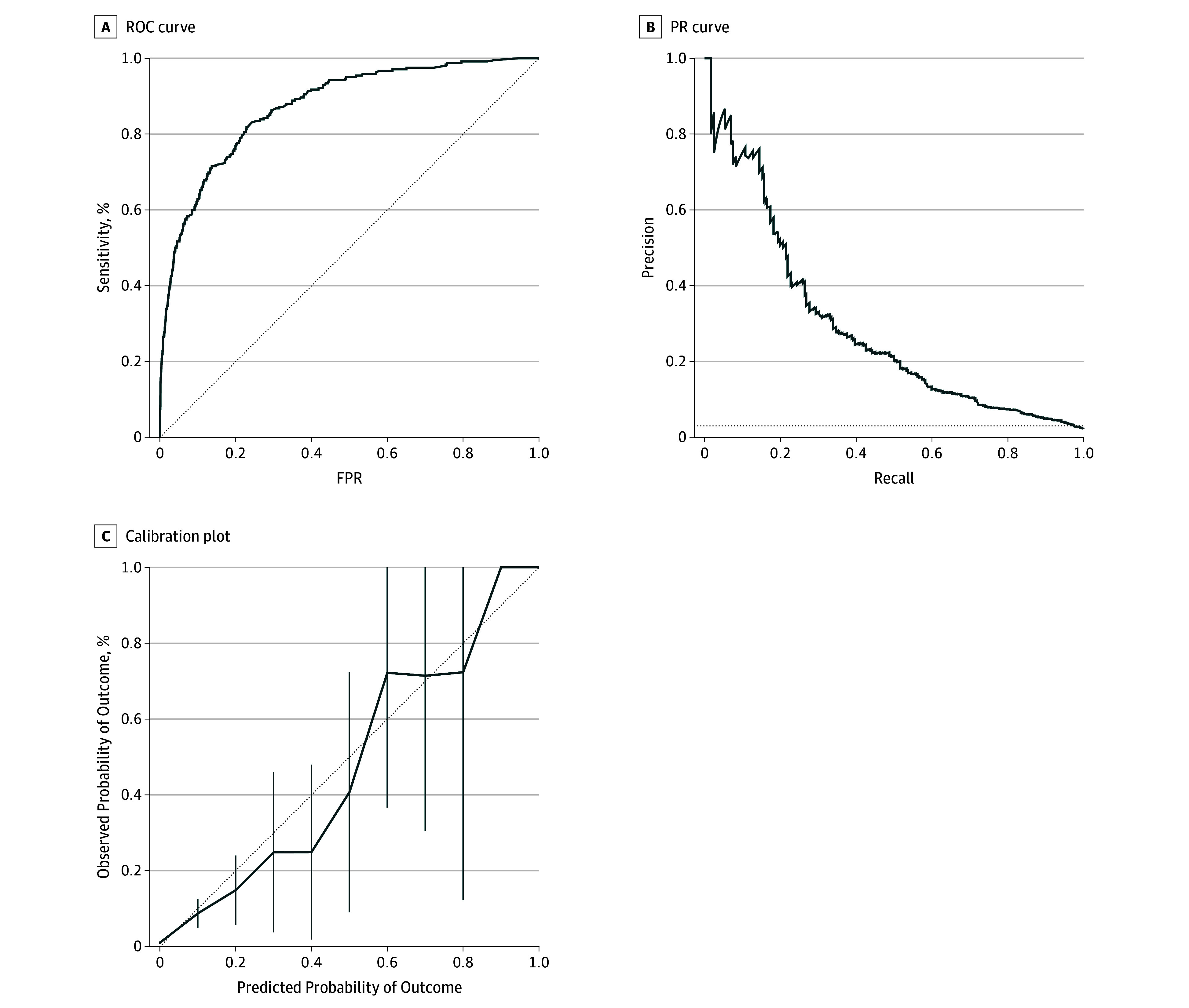
Receiver Operating Characteristic (ROC) Curve, Precision Recall (PR) Curve, and Calibration Plot for 2014 to 2015 Test Set A, The area under the curve was 0.873. FPR indicates false-positive rate. B, The area under the curve was 0.288. C, The error bars indicate 95% confidence intervals.

In Figure 1, the ROC and PR curves for the training test set demonstrate that the model performed well across risk threshold values, and the calibration plot demonstrates that mortality risk predicted by the model approximated observed mortality rates. The ROC and PR curves for the other evaluations are shown in eFigure 2 in the Supplement.

Table 3 shows metrics that are most relevant to operational stakeholders in the 2014 to 2015 test set and assumes a theoretical admission volume of 100 patients per day. At this volume, alerts triggered at or above a threshold of 0.04 would be 61% sensitive and 91% specific and would result in 11.9 alerts per day. Of all fired alerts, 1.5 would be true alerts and 10.4 would be false alerts. At this risk threshold, the workup to detection ratio is 7.9. As shown in Table 3, these metrics changed with different risk thresholds.

**Table 3.** Operational Performance Metrics for Various Model Thresholds for the Entire 2014 to 2015 Test Set, Assuming a Theoretical Admission Volume of 100 Patients per Day

Threshold	Sensitivity	Specificity	PPV	Alerts, No./d		
				Total	False	True
0.01	0.88	0.66	0.05	39.9	37.8	2.1
0.02	0.76	0.81	0.08	23.3	21.5	1.8
0.03	0.68	0.88	0.11	15.3	13.6	1.7
0.04	0.61	0.91	0.12	11.9	10.4	1.5
0.05	0.57	0.93	0.15	9.1	7.7	1.4
0.06	0.54	0.95	0.18	7.4	6.1	1.3
0.07	0.52	0.95	0.19	6.5	5.3	1.3
0.08	0.50	0.96	0.21	5.8	4.5	1.2
0.09	0.48	0.96	0.22	5.2	4.1	1.2
0.10	0.44	0.97	0.22	4.8	3.7	1.1
0.11	0.43	0.97	0.24	4.4	3.4	1.0
0.12	0.41	0.97	0.24	4.1	3.1	1.0
0.13	0.39	0.98	0.26	3.7	2.7	1.0
0.14	0.39	0.98	0.27	3.5	2.6	0.9
0.15	0.36	0.98	0.27	3.2	2.3	0.9
0.16	0.35	0.98	0.28	3.1	2.2	0.9
0.17	0.34	0.98	0.30	2.8	2.0	0.8
0.18	0.33	0.98	0.32	2.6	1.7	0.8
0.19	0.31	0.99	0.32	2.4	1.6	0.8
0.20	0.29	0.99	0.33	2.2	1.5	0.7
0.21	0.28	0.99	0.33	2.0	1.4	0.7
0.22	0.28	0.99	0.35	1.9	1.3	0.7
0.23	0.27	0.99	0.36	1.8	1.2	0.7
0.24	0.26	0.99	0.38	1.7	1.1	0.6
0.25	0.26	0.99	0.41	1.5	0.9	0.6

Abbreviation: PPV, positive predictive value.

The eTable in the Supplement shows model performance across patient subpopulations in the 2014 to 2015 test set. The risk threshold was selected to achieve a PPV of 20% across the entire 2014 to 2015 retrospective test set. The threshold of 0.075 resulted in an operationally manageable level of 6 total alerts per day (for 100 admissions per day), with 5 false alerts and 1 true alert. Model sensitivity, specificity, and PPV varied across patient subpopulations segmented by information missingness, age, race, sex, admission types, and admission sources.

**Figure 2.**
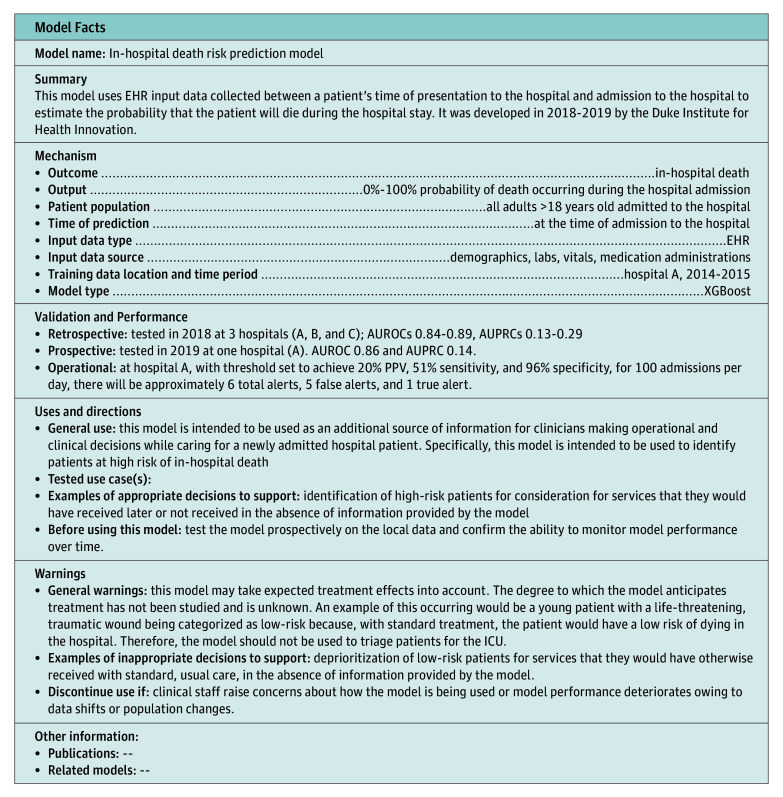
Model Fact Sheet to Communicate Important Model Information to Clinical Partners AUPRC indicates area under the precision recall curve; AUROC, area under the receiver operating characteristic curve; EHR, electronic health record; ICU, intensive care unit; and PPV, positive predictive value.

eFigure 3 in the Supplement shows an Apache Superset dashboard that was developed to display patient risk scores to clinicians to support the development of workflows using model output. The patient list can be filtered by time range to include patients admitted in the last day, last week, last month, and last year and also includes an option for searching for a specific patient medical record number. The dashboard was built from a curated database that automatically updates daily from the EHR reporting database. eFigure 4 in the Supplement shows the framework used to develop clinical workflows to be supported by model output. Figure 2 shows a model fact sheet, which summarizes important model information in a style similar to an over-the-counter drug label. The fact sheet is intended to guide use of the model by clinical end users who may not be familiar with the inner workings of the machine learning model.

## Discussion

We developed a machine learning model to predict in-hospital mortality at the time of admission for all adult patients using data that are readily accessible in the EHR. We retrospectively evaluated the model to assess temporal and external geographic generalizability and we prospectively evaluated model performance using a data pipeline that is integrated with our operational EHR. The model uses off-the-shelf software packages,^25,26,27^ was successfully integrated into a production EHR, and can be similarly integrated at other sites.

To make the model implementable at a system level, the model was trained on all adult patients using data elements commonly available across sites. The model only uses information from the current encounter without prehospital information, meaning that model outputs are accurate for patients who present for the first time to a health care setting. We limited the end point of data to time of inpatient admission order because this represented a single point for which we could generate a model prediction that would be immediately actionable to an admitting team. Inpatient admitting teams often care for patients for multiple days, and our clinical partners and operational stakeholders wanted to improve the rate at which goals of care conversations occur shortly after admission. Moreover, this is a commonly used point to predict mortality end points.^7,8,9,10,14^

There are previously published^7,8,9,10,11^ models that predict inpatient mortality at the time of admission. Rajkomar et al^7^ used deep learning in combination with tokenized data processing built on Fast Healthcare Interoperability Resources to train site-specific mortality models. The model ingests all data available for a patient up until the time of prediction and achieved an AUROC of 0.90. The model does not require manual curation of features, which we have shown can cost more than $200 000 per application.^18^ However, implementing this type of model requires potentially more substantial investment, including mapping all EHR data to Fast Healthcare Interoperability Resources resources and more than 200 000 hours of graphical processing unit training time.^27^ To our knowledge, the model has not yet been integrated into an operational EHR and evaluated prospectively. Tabak et al^8^ used logistic regression and conversion to an integer-based score (ALaRMS) to achieve an AUROC of 0.87. The model ingests demographic data and initial laboratory test results across 23 numeric tests to predict inpatient mortality at the time of admission. The model was developed using data from 70 hospitals in the United States and has been externally validated by an independent team in Switzerland.^11^ In addition to externally validating ALaRMS, Nakas et al^11^ developed decision tree and neural network models that ingested the same demographic and laboratory test result inputs, and both models achieved AUROCs of 0.91. Schwartz et al^9^ used penalized logistic regression to develop a model that ingests demographic characteristics, initial laboratory test results during a hospital encounter, and prehospital comorbidities to predict inpatient mortality. The model achieved an AUROC of 0.857 and has not been externally or prospectively validated. Escobar et al^10^ used logistic regression to develop a model that ingests laboratory test results, vital signs, neurological status, comorbidities, primary condition, admission venue, code status, and demographic data to predict inpatient mortality at the time that the patient is transferred to an inpatient bed. The model achieved an AUROC of 0.883 and has not been externally or prospectively evaluated. Several other closely related models predicting deterioration and ICU transfer have been retrospectively evaluated and integrated into production EHRs.^12,13,14,15^ To our knowledge, our model is the first machine learning model that predicts in-hospital mortality to be prospectively validated after integration into an operational EHR.

Prospective validation on operational data is an important first step in assessing the real-world performance of machine learning models. Ensuring that a model continues to perform well during a silent period sets the stage for integration into clinical workflows and evaluation of clinical and operational impact. Unfortunately, few machine learning models have been evaluated prospectively using real-world EHR data,^32^ although there have been several recent validations of medical imaging models in the real world.^33,34,35^ Our successful prospective validation is the first described in the literature for inpatient mortality prediction using a production EHR. The results in Table 2 show that model performance prospectively (AUROC = 0.86) almost exactly matched model performance retrospectively across the original test set (AUROC = 0.87) and the 2018 cohort (AUROC = 0.85). The AUPRC in the prospective cohort (0.14) was lower than the retrospective AUPRC at the same hospital in 2014 to 2015 (0.29), which in part may have been due to the 47% reduction in prevalence of in-hospital mortality in the prospective cohort compared with the retrospective cohort (1.6% vs 3.0%). Of note, when prevalence rates differ across cohorts, it can be challenging to compare and interpret differences in AUPRC.^36^ In the 2018 temporal and external validation cohorts, if the model were implemented in each setting at a threshold that corresponds to a sensitivity (recall) of 60%, the PPV is 9.8% in hospital A, 8.0% in hospital B, and 9.1% in hospital C. In the prospective validation cohort, using a threshold that corresponds to a sensitivity of 60% would lead to a PPV of 8.5%. Considering these important differences, sites wishing to implement this model may need to conduct localized prospective validations and set thresholds that best align with local performance. Overall, taking into account differences in in-hospital mortality rates between the 2 cohorts, these results suggest that model discrimination remained stable and that there would be a slight increase in number of false-positive alerts when evaluated prospectively on operational data.

Similarly, external validation results suggest stability in model performance when run on data from nontraining sites and outside of the training time period. The AUROC in hospitals B and C in 2018 (0.89 and 0.84, respectively) are consistent with the AUROC for training hospital A in 2014 to 2015 and 2018 (0.87 and 0.84, respectively). Model stability over time and across settings suggests that the model may not need to be retrained, updated, or replaced as frequently. Furthermore, the possibility that models trained on data from 1 academic hospital in a health system can perform well at community hospitals in the same system may enable health systems to scale the clinical benefit realized from a single model without needing to rebuild site-specific models. However, differences in outcome prevalence rates between sites and time periods must be examined and continually monitored. Threshold values may need to be adjusted as the PPV of alerts changes with outcome prevalence rate and as the number of alerts changes with admission volume.

In addition to performing well prospectively and on external sites, for machine learning models to improve patient care, they must also change behavior.^37^ This requires improving decisions, which in the hospital are often made by physicians. During our efforts to develop workflows for this model with our clinical partners, we used a framework of identifying important decisions, specifying the decision-maker, understanding the point at which the decision is made, verifying that there is an opportunity to improve, and confirming that the improvement can be measured (eFigure 4 in the Supplement). The dashboard (eFigure 3 in the Supplement), which shows a list of patient risk scores generated on a daily basis, coupled with the model fact sheet (Figure 2) helped clinicians understand the types of decisions that the model should and should not be used to support. Ultimately, this aided the process of identifying use cases that can be tested and measured.

There is growing consensus that machine learning model reporting needs to be more standardized for a clinical audience^38^ as well as more transparent to end users.^39^ To our knowledge, the model fact sheet displayed in Figure 2 is the first formal effort in health care to distill important facts about how a model is developed and how a model should be used into 1 page. The fact sheet has undergone several iterations incorporating feedback from internal stakeholders. The need for such an artifact emerged after conversations with end users about the various ways the model output could inform clinical decision-making. One especially troubling use of the model is called out in the model fact sheet. The model should not be used to prioritize patients for admission to the ICU or to limit care delivery. The model is built to predict risk of mortality at any point during the encounter, and patients may receive life-saving therapies during an encounter that decrease risk of inpatient mortality. A study from 2015 describes how asthma was identified by a machine learning model as protective against death for patients admitted to a hospital with pneumonia.^40^ The reason was that patients with asthma were admitted directly to an ICU and received appropriate care that was protective against inpatient mortality. If the model were prospectively implemented and incorporated into clinical decision making at the time of admission, it is not difficult to imagine patients with asthma being deprioritized for ICU admission. The downstream consequences are significant. The model should only be used to identify high-risk patients for consideration for services that they would have received later or not received at all in the absence of information provided by the model. The first use case that we plan to evaluate is identification of patients for palliative care consultation. The model fact sheet includes sections that are currently empty that we hope to populate as the evidence base for the current model grows.

Many challenges were encountered in prospectively evaluating the model. First and foremost, the technology infrastructure required to run models in real time had to be built. Fortunately, at our institution, such an infrastructure to automatically curate EHR data was already in place, using native functionality in addition to custom developed technologies.^30^ Second, there were many differences in data element names between the retrospective training data and prospective, live EHR data. This was due to changes in the EHR data structure over time that are not necessarily visible to the clinical end user. Because of this, extensive mapping of variable names from the old retrospective data to the new prospective data was required. Third, differences in software versions between the computational research environment in which the model was trained and the production environment in which the model was run had to be reconciled.

### Limitations

Our study has several potential limitations. First, we excluded patients younger than eighteen years, and therefore our model will not be able to be used on pediatric patients. Second, it is rare for institutions to possess the technology infrastructure required to deploy models in a clinical setting on live EHR data, and the scalability of this technical challenge is not addressed by our methods. The approach is scalable and effort and investment would likely be required by sites interested in adopting a similar approach. Third, the prospective validation was a silent period and did not include any interventions. While we demonstrate that the model prediction accuracy is maintained prospectively, it is unknown whether these predictions will actually improve clinical or operational outcomes. Future work will evaluate the implementation of a clinical workflow. Fourth, although we demonstrate external generalizability to community hospitals 4 and 30 miles from a flagship academic hospital, additional research is required to assess generalizability to more distant regions. Fifth, based on related work by others^5^ predicting inpatient mortality as well as prior internal experience predicting sepsis,^41^ further performance gains may be achievable using deep learning. Sixth, the decision to generate a single prediction at the time of order placement for inpatient admission and only use data prior to that point is subject to bias as to when the emergency department clinician places the order. The subanalysis in eTable 1 in the Supplement further demonstrates that the model performs worse for patients with fewer data points collected prior to inpatient admission (sensitivity of 33% and specificity of 99% for patients with missing vital signs, laboratory test results, or medication data vs sensitivity of 57% and specificity of 94% for patients without any missing data). Seventh, the current study does not provide insight into the value of any particular input variable. Prior literature^7,8,10^ suggests that comorbidities, vital signs, and laboratory test results each add value to inpatient mortality prediction models, and further research is required to optimize model performance with limited inputs. Eighth, a small percentage of patients with multiple encounters were represented both in the training and validation sets. On further inspection, we found that of the patients in the hospital A training set, 4.5% were in the hospital A test set, 1.2% were in the hospital B test set, and 0.4% were in the hospital C test set. As a result, model performance in validation sets may be biased by multiple predictions of the same patient with multiple encounters. In addition, further research is needed to understand how the model output should be interpreted and used in the clinical setting. For example, this study does not elucidate to what extent the model has learned treatment effects, and without careful instruction for how to interpret model output, clinicians may underestimate in-hospital mortality risk for patients with dangerous conditions that would usually receive intensive treatment. The goal of our model fact sheet is to highlight the recommended use cases as well as warn against use cases that may limit access to needed therapies in the inpatient setting. We hope the model fact sheet serves as a template for other teams conducting related work.

## Conclusions

Taken together, the findings in this study provide encouraging support that machine learning models to predict in-hospital mortality can be implemented on live EHR data with prospective performance matching performance seen in retrospective evaluations of highly curated research data sets. This silent-period validation sets the stage for integration into clinical workflows. The benefit to cost ratio of developing and deploying models in clinical settings will continue to increase as commonly available EHR data elements are more effectively used and opportunities to scale models externally are identified. Further research is required to understand how to best integrate these models into the clinical workflow, identify opportunities to scale, and quantify the impact on clinical and operational outcomes.

## Notes

Supplement. **eFigure 1.** Data Elements and Data Features **eFigure 2.** ROC and PR Curves for Retrospective and Prospective Evaluations **eTable.** Model Performance for Various Subpopulations, Using Risk Threshold That Achieves 20% PPV and 51% Sensitivity for the Entire 2014-2015 Test Set **eFigure 3.** Apache Superset Dashboard Used to Support Development of Workflows **eFigure 4.** Framework for Developing Clinical Workflows to Be Supported by Model Output
