# Supplementary material for: Prospective and External Evaluation of a Machine Learning Model to Predict In-Hospital Mortality of Adults at Time of Admission
Source: JAMA Netw Open. 2020 Feb 7;3(2):e1920733. doi: 10.1001/jamanetworkopen.2019.20733 (PMC12068827; doi:10.1001/jamanetworkopen.2019.20733)
Supplement: Supplement. — eFigure 1. Data Elements and Data Features eFigure 2. ROC and PR Curves for Retrospective and Prospective Evaluations eTable. Model Performance for Various Subpopulations, Using Risk Threshold That Achieves 20% PPV and 51% Sensitivity for the Entire 2014-2015 Test Set eFigure 3. Apache Superset Dashboard Used to Support Development of Workflows eFigure 4. Framework for Developing Clinical Workflows to Be Supported by Model Output [file jamanetwopen-e1920733-s001.pdf]

Supplementary Online Content

Brajer N, Cozzi B, Gao M, et al. Prospective and external evaluation of a machine learning model to predict in-hospital mortality of adults at time of admission. *JAMA Netw Open*. 2020;3(2):e1920733. doi:10.1001/jamanetworkopen.2019.20733

eFigure 1. Data Elements and Data Features
eFigure 2. ROC and PR Curves for Retrospective and Prospective Evaluations
eTable. Model Performance for Various Subpopulations, Using Risk Threshold That Achieves 20% PPV and 51% Sensitivity for the Entire 2014-2015 Test Set
eFigure 3. Apache Superset Dashboard Used to Support Development of Workflows
eFigure 4. Framework for Developing Clinical Workflows to Be Supported by Model Output

This supplementary material has been provided by the authors to give readers additional information about their work.

© 2020 Brajer N et al. *JAMA Network Open.*

eFigure 1. Data elements and data features

**Data element type**	**Data element name**
Lab	blood culture
Lab	glucose
Lab	CK_MB
Lab	platelet
Lab	hct
Lab	WBC
Lab	creatinine
Lab	BUN
Lab	potassium
Lab	sodium
Lab	albumin
Lab	bilirubin
Lab	ALT
Lab	AST
Lab	creatine_kinase
Lab	magnesium
Lab	bands
Lab	INR
Lab	bicarb_ven
Lab	PCO2_ven
Lab	pH_ven
Lab	lactate
Lab	bicarb_art
Lab	PO2_art
Lab	PCO2_art
Lab	pH_art
Lab	ESR
Lab	d_dimer
Lab	CRP
Lab	fibrinogen
Lab	LDH
Lab	ammonia
Lab	trop_t
Medication administration	heparin
Medication administration	fluids
Medication administration	opioid
Medication administration	abx
Medication administration	steroids
Medication administration	insulin
Medication administration	benzo
Medication administration	immuno
Medication administration	chemo
Medication administration	vasopressor
Vital sign	mental_status
Vital sign	weight
Vital sign	supp_o2
Vital sign	diastolic_BP
Vital sign	systolic_BP
Vital sign	HR
Vital sign	pulse_ox
Vital sign	RR
Vital sign	temp
Demographic	Admission source
Demographic	Admission type
Demographic	Age
Demographic	Sex
Demographic	Race

**Model Features**			
admission_source	max_hct	var_ck_mb	count_wbc
admission_type	max_inr	var_creatine_kinase	min_diastolic_bp
sex	max_lactate	var_creatinine	min_hr
race	max_ldh	var_crp	min_pulse_ox
age	max_magnesium	var_d_dimer	min_rr
min_albumin	max_pco2	var_esr	min_systolic_bp
min_alt	max_ph	var_fibrinogen	min_temp
min_ammonia	max_platelet	var_glucose	max_diastolic_bp
min_ast	max_po2	var_hct	max_hr
min_bands	max_potassium	var_inr	max_pulse_ox
min_bicarb	max_sodium	var_lactate	max_rr
min_bilirubin	max_trop_t	var_ldh	max_systolic_bp
min_bun	max_wbc	var_magnesium	max_temp
min_ck_mb	mean_albumin	var_pco2	mean_diastolic_bp
min_creatine_kinase	mean_alt	var_ph	mean_hr
min_creatinine	mean_ammonia	var_platelet	mean_pulse_ox
min_crp	mean_ast	var_po2	mean_rr
min_d_dimer	mean_bands	var_potassium	mean_systolic_bp
min_esr	mean_bicarb	var_sodium	mean_temp
min_fibrinogen	mean_bilirubin	var_trop_t	mean_weight
min_glucose	mean_bun	var_wbc	var_diastolic_bp
min_hct	mean_ck_mb	count_albumin	var_hr
min_inr	mean_creatine_kinase	count_alt	var_pulse_ox
min_lactate	mean_creatinine	count_ammonia	var_rr
min_ldh	mean_crp	count_ast	var_systolic_bp
min_magnesium	mean_d_dimer	count_bands	var_temp
min_pco2	mean_esr	count_bicarb	count_diastolic_bp
min_ph	mean_fibrinogen	count_bilirubin	count_hr
min_platelet	mean_glucose	count_blood_culture	count_mental_status
min_po2	mean_hct	count_bun	count_pulse_ox
min_potassium	mean_inr	count_ck_mb	count_rr
min_sodium	mean_lactate	count_creatine_kinase	count_supp_o2
min_trop_t	mean_ldh	count_creatinine	count_systolic_bp
min_wbc	mean_magnesium	count_crp	count_temp
max_albumin	mean_pco2	count_d_dimer	count_weight
max_alt	mean_ph	count_esr	count_abx
max_ammonia	mean_platelet	count_fibrinogen	count_benzo
max_ast	mean_po2	count_glucose	count_chemo
max_bands	mean_potassium	count_hct	count_fluids
max_bicarb	mean_sodium	count_inr	count_heparin
max_bilirubin	mean_trop_t	count_lactate	count_immuno
max_bun	mean_wbc	count_ldh	count_insulin
max_ck_mb	var_albumin	count_magnesium	count_opioid
max_creatine_kinase	var_alt	count_pco2	count_steroids
max_creatinine	var_ammonia	count_ph	count_vasopressor
max_crp	var_ast	count_platelet	
max_d_dimer	var_bands	count_po2	
max_esr	var_bicarb	count_potassium	
max_fibrinogen	var_bilirubin	count_sodium	
max_glucose	var_bun	count_trop_t	

© 2020 Brajer N et al. *JAMA Network Open.*

eFigure 2. ROC and PR curves for retrospective and prospective evaluations

Retrospective Hospital A 2018	Retrospective Hospital B 2018	Retrospective Hospital C 2018	Prospective Hospital A 2019

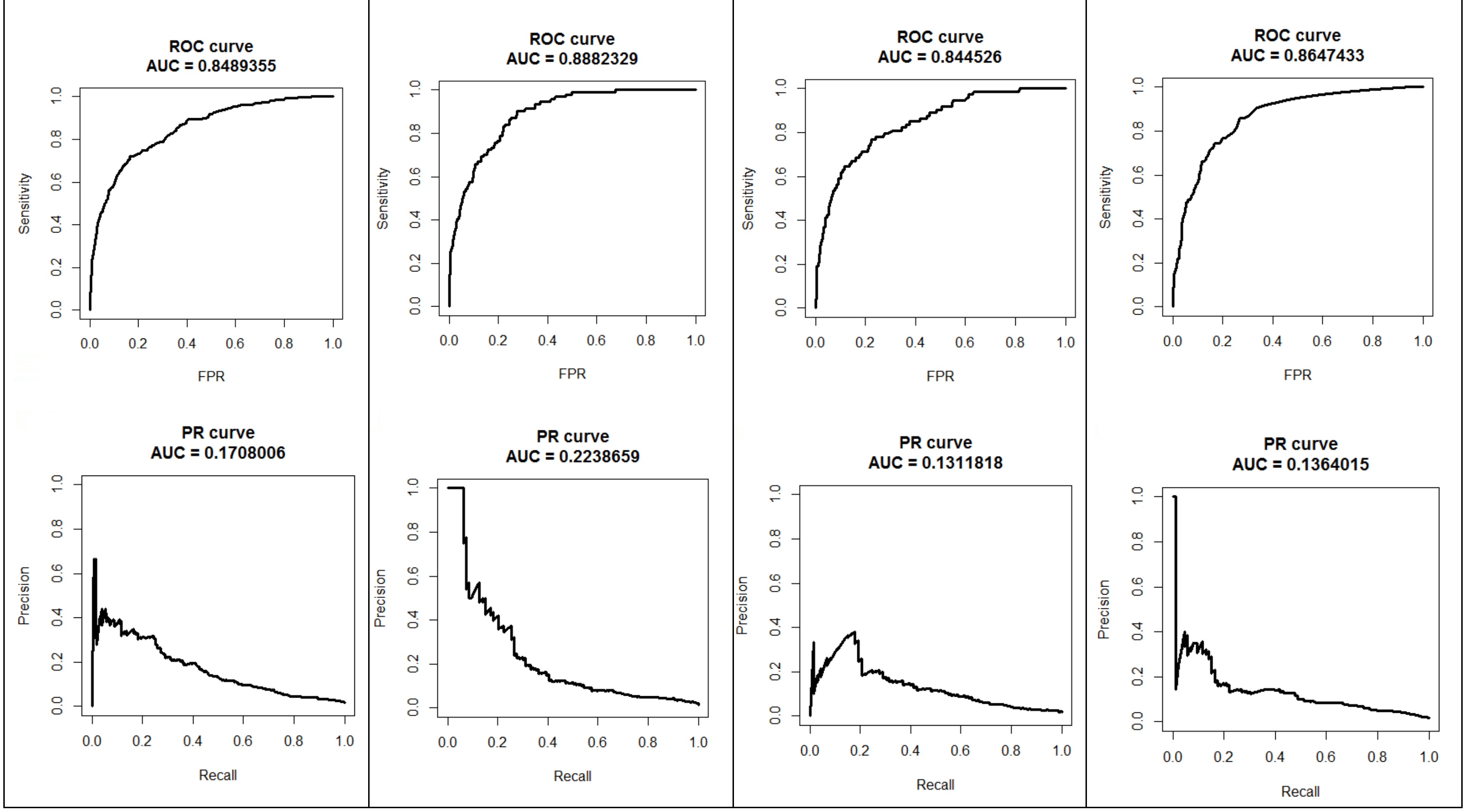

© 2020 Brajer N et al. *JAMA Network Open.*

**eTable.** Model performance for various subpopulations, using risk threshold that achieves 20% PPV and 51% sensitivity for the entire 2014-2015 test set					
	**Count (No.)**	**Deaths (No.)**	**Sensitivity**	**Specificity**	**PPV**
Vitals, labs, & medication data					
Present	8117	190	0.57	0.94	0.18
Missing	3314	51	0.33	0.99	0.29
Age > 65					
False	7309	94	0.50	0.96	0.15
True	4122	147	0.53	0.93	0.22
Admission type					
Emergency	5149	193	0.64	0.90	0.19
Elective	3936	22	0.00	1.00	0.00
Urgent	2345	26	0.04	0.99	0.06
Sex					
Female	6195	101	0.53	0.96	0.19
Male	5234	140	0.51	0.94	0.19
Race					
Black	3291	70	0.60	0.95	0.20
White	7240	161	0.48	0.95	0.19
Admission source					
Home or Non-Health Care Facility Point of Origin	10254	177	0.44	0.95	0.14
Clinic or Physician Office	867	28	0.46	0.97	0.33
Transfer from Another Health Care Facility	124	5	0.80	0.91	0.27
Transfer from a Skilled Nursing Facility (SNF), ICF or ALF	89	29	0.97	0.43	0.45

© 2020 Brajer N et al. *JAMA Network Open.*

eFigure 3. Apache Superset dashboard used to support development of workflows

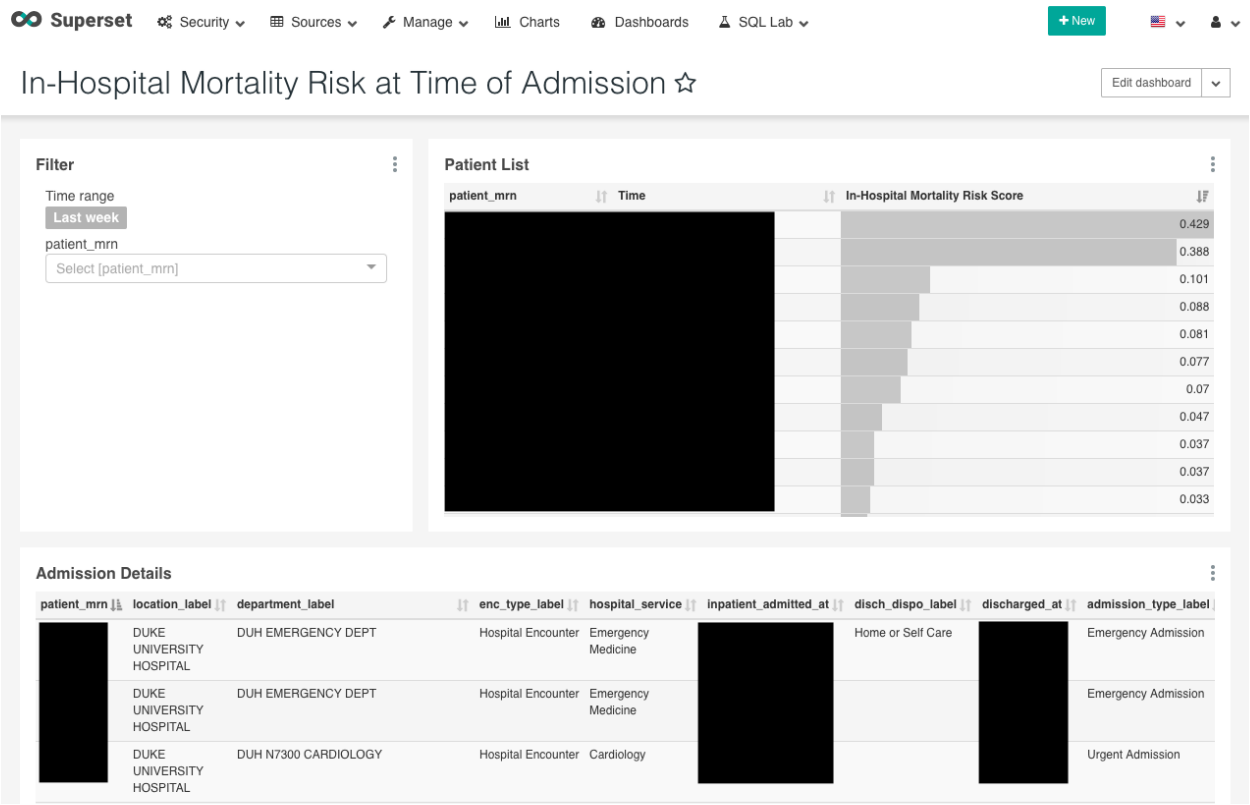

eFigure 4. Framework for developing clinical workflows to be supported by model output

	Workflow Idea	Decision maker	Decision	Time Decision is Made	Metric to Shift	Metric baseline	Opportunity to improve?
1							
2							
3							

© 2020 Brajer N et al. *JAMA Network Open.*
